# Leadership and Employees’ Mental Health Under Disruption: A Scoping Review and Critical Interpretive Synthesis

**DOI:** 10.3390/ejihpe16090138

**Published:** 2026-09-11

**Authors:** Carlos Santiago-Torner, Elisenda Tarrats-Pons, José-Antonio Corral-Marfil

**Affiliations:** 1 REDEES Research Group, Faculty of Economics and Business, International University of La Rioja (UNIR), 26006 Logroño, Spain; carlos.santiago-externo@unir.net; 2 Department of Economics and Business, University of Vic—Central University of Catalonia (UVic-UCC), 08500 Vic, Spain; elisenda.tarrats@uvic.cat

**Keywords:** leadership in crisis, employee burnout, psychological safety, trust, psychological capital, emotional regulation

## Abstract

This scoping review examines how leadership shapes employees’ mental health in disruptive, uncertain organizational contexts between 2020 and 2025. Following PRISMA-ScR guidelines, a systematic search across Web of Science, Scopus, PubMed, and PsycINFO yielded 43 eligible sources (30 empirical, 13 conceptual/theoretical). Data were extracted and synthesized through critical interpretive synthesis. Findings indicate that transformational, ethical, servant, inclusive, and health-oriented leadership are associated with favorable well-being outcomes and with psychosocial resources including psychological safety, trust, and psychological capital. Conversely, authoritarian, toxic, destructive, and laissez-faire leadership are associated with burnout, psychological distress, organizational silence, and turnover intentions. Building on these observed associations, the interpretive synthesis generates three theoretically plausible propositions: leaders’ emotional regulation may condition the protective potential of prosocial leadership; resource depletion under disruption may contribute to the intensification of dysfunctional leadership; and psychological safety, trust, and psychological capital may operate as interconnected mechanisms linking leadership with emotional exhaustion and resilience. These propositions represent synthesis-generated relationships requiring direct empirical testing rather than effects established by the reviewed evidence. Synthesizing these insights, this review proposes a dynamic emotional regulation framework of leadership under disruption. Rather than viewing leadership through static styles, the framework conceptualizes it as a relational, context-sensitive process whose implications for occupational mental health depend on psychosocial and contextual conditions.

## 1. Introduction

Since 2020, organizations have operated under overlapping disruptions that have transformed not only how work is organized but also how it is emotionally experienced. The COVID-19 pandemic, geopolitical instability, economic uncertainty, labor precarity, accelerated digitalization, and the expansion of hybrid and remote work have intensified ambiguity, emotional demands, and adaptation pressures (Khan et al., 2020; Rudolph et al., 2020). These disruptions have challenged coordination and decision-making while exposing the psychological vulnerability of both employees and leaders. Leadership has consequently become a central psychosocial process through which organizations interpret uncertainty, shape emotional climates, preserve trust, and sustain collective functioning under strain (Lester et al., 2024; Riggio & Newstead, 2023).

Leadership research has traditionally emphasized performance, motivation, strategic alignment, and decision-making effectiveness. Evidence generated during and after the pandemic, however, increasingly shows that leadership also has direct and indirect implications for occupational mental health. Leaders who provide emotional support, ethical guidance, relational stability, and psychologically safe conditions may buffer stress, uncertainty, and exhaustion. Conversely, dysfunctional leadership can intensify emotional strain, distrust, withdrawal, burnout, and organizational fragmentation (De Clercq & Belausteguigoitia, 2021; Labrague, 2024; P. Li et al., 2024; Vonderlin et al., 2023). Leadership therefore needs to be understood not only as a mechanism of organizational influence but also as a psychosocial and emotionally embedded process operating within particular conditions of work.

A growing empirical literature associates transformational, ethical, servant, inclusive, and health-oriented leadership with engagement, resilience, psychological safety, commitment, and well-being (Ahmed et al., 2023; Klebe et al., 2021; Kloutsiniotis et al., 2022; X. Li & Peng, 2022). In contrast, authoritarian, toxic, destructive, and laissez-faire leadership have been associated with emotional exhaustion, psychological distress, silence, turnover intentions, and deteriorating organizational climates (Asim et al., 2021; Farghaly Abdelaliem & Abou Zeid, 2023; Nunes & Palma-Moreira, 2024; Palvimo et al., 2023; Zheng et al., 2025). Systematic reviews and meta-analytical evidence reinforce the relevance of these relationships, particularly in high-pressure sectors such as healthcare, hospitality, and service work (Labrague, 2024; P. Li et al., 2024; Wong et al., 2025).

Despite this progress, the evidence remains heterogeneous and fragmented in at least three respects. First, leadership approaches are frequently examined as discrete categories, with insufficient attention to the emotional processes through which their effects may change under disruption. Second, leadership–health relationships are commonly investigated through isolated mediators or outcomes rather than integrated psychosocial pathways. Third, existing evidence does not sufficiently explain why adaptive forms of leadership may lose effectiveness under sustained pressure or why dysfunctional behaviors may intensify as uncertainty and resource constraints increase. This fragmentation extends across leadership constructs, organizational settings, methodological designs, psychosocial mechanisms, and mental health outcomes, making direct comparison and theoretical integration particularly difficult.

This heterogeneity provides the central rationale for a scoping review. The literature spans cross-sectional and longitudinal research, intervention studies, qualitative evidence, systematic reviews, meta-analyses, and conceptual contributions distributed across organizational psychology, management, and occupational health. Mapping this evidence is therefore necessary to establish its scope and characteristics, identify recurring and underexplored relationships, and clarify how leadership, contextual pressure, psychosocial mechanisms, and employee mental and emotional health have been connected across different forms of evidence. At the same time, critical interpretive synthesis enables the mapped evidence to be examined beyond descriptive categorization by identifying convergences, contradictions, explanatory mechanisms, and theoretical gaps.

A central tension concerns the assumed protective character of prosocial leadership. Transformational, ethical, servant, inclusive, and health-oriented approaches are generally associated with trust, psychological safety, resilience, and well-being. Yet leaders themselves may become emotionally overloaded, depleted, or dysregulated under prolonged uncertainty, conflicting demands, and insufficient organizational support. Their capacity to sustain protective behaviors may therefore weaken. Conversely, dysfunctional leadership is commonly attributed to stable traits or destructive orientations, although crisis-related resource depletion and emotional dysregulation may also amplify authoritarian, toxic, destructive, or avoidant behaviors. A simple distinction between positive and negative leadership styles may consequently obscure the contextual and regulatory conditions under which leadership protects or undermines employee health.

A second unresolved issue concerns the mechanisms connecting leadership with health outcomes. Psychological safety, trust, organizational justice, ethical climate, resilience, and psychological capital recur throughout the literature but are frequently examined separately. This fragmentation restricts understanding of how leadership may influence emotional exhaustion and resilience through interconnected psychosocial processes. Ibero-American research further illustrates the contextual relevance of these mechanisms by showing how ethical leadership moderates the consequences of selfish ethical climates in teleworking environments, how positive occupational mental health and organizational socialization predict commitment, and how psychological safety mediates relationships involving transformational leadership and organizational climate (Fajardo Castro et al., 2025; Orozco-Solis et al., 2022; Santiago-Torner, 2023). Recent evidence also confirms that ethical leadership is closely associated with reduced emotional exhaustion in virtual work settings (Santiago-Torner et al., 2024a). These findings reinforce the need to map relational and contextual mechanisms alongside leadership orientations and health outcomes.

Against this background, the objective of this scoping review is to map and critically integrate evidence published between 2020 and 2025 on how leadership relates to employees’ mental and emotional health under organizational disruption, uncertainty, and sustained pressure. Specifically, the review examines the leadership approaches represented in the literature, the mental and emotional health outcomes associated with them, the psychosocial mechanisms through which these relationships are explained, and the contextual conditions that may strengthen, weaken, or alter these relationships. Through critical interpretive synthesis, the review further seeks to identify recurrent explanatory relationships capable of supporting theoretical integration and future empirical research.

Three analytical propositions were subsequently developed from recurrent patterns, tensions, and explanatory gaps identified through the synthesis. Proposition 1 (P1) proposes that leaders’ emotional regulation capacity may constitute a boundary condition shaping the extent to which prosocial leadership approaches, particularly ethical, transformational, servant, inclusive, and health-oriented leadership, remain protective under disruption. Proposition 2 (P2) proposes that resource depletion and emotional dysregulation under sustained pressure may contribute to the persistence or intensification of dysfunctional leadership, including toxic, authoritarian, destructive, and laissez-faire leadership, beyond dispositional explanations alone. Proposition 3 (P3) proposes that psychological safety, trust, and psychological capital may constitute interconnected, rather than necessarily sequential, psychosocial mechanisms linking leadership with emotional exhaustion and resilience during periods of systemic disruption.

These propositions are not presented as predefined hypotheses tested through the review or as definitive causal claims. Rather, they represent theoretically grounded analytical propositions generated through the interpretive synthesis and intended for subsequent empirical examination. They distinguish relationships directly supported across the reviewed literature from higher-order explanatory relationships generated through synthesis. Their function is to organize fragmented evidence into theoretically meaningful relationships among leadership orientation, emotional regulation, psychosocial mechanisms, and mental health outcomes, while establishing a research agenda for organizational psychology and occupational health.

By integrating the mapped evidence through critical interpretive synthesis, this scoping review develops a dynamic emotional regulation framework of leadership under disruption. The proposed framework conceptualizes leadership as a relational and context-sensitive process in which employee health is associated with psychosocial mechanisms, leaders’ regulatory resources, and conditions of organizational pressure. The study therefore contributes to organizational psychology by clarifying potential relational mechanisms connecting leadership with employee functioning and to occupational health by positioning mental and emotional health as central outcomes through which leadership effectiveness should be understood.

## 2. Materials and Methods

### 2.1. Design and Review Logic

This study adopts a scoping review design combined with critical interpretive synthesis to examine how leadership shapes employees’ mental and emotional health in organizational contexts characterized by disruption, uncertainty, and sustained pressure. The scoping review design was selected because the literature is heterogeneous in its theoretical foundations, methodological approaches, leadership constructs, occupational settings, and mental health outcomes. Such heterogeneity requires a review strategy capable of mapping the breadth and characteristics of available evidence while preserving meaningful conceptual and contextual differences. Critical interpretive synthesis complements this mapping function by enabling comparison of divergent findings, identification of conceptual tensions, and development of higher-order theoretical explanations (Ferrari, 2015; Gilson & Goldberg, 2015; Greenhalgh et al., 2018).

This combined design is appropriate because the evidence base encompasses cross-sectional and longitudinal studies, interventions, qualitative investigations, systematic reviews, meta-analyses, theoretical contributions, and integrative frameworks. Rather than reducing heterogeneous sources to a common effect metric, the review examines how different forms of evidence converge, diverge, or qualify assumptions about leadership and occupational mental health. This orientation is consistent with problematization-based approaches to theory development, which question established assumptions when prevailing frameworks provide only partial explanations of complex organizational phenomena (Alvesson & Sandberg, 2011).

The review was conducted and reported in accordance with the PRISMA extension for Scoping Reviews (PRISMA-ScR), emphasizing transparent reporting of eligibility criteria, information sources, search procedures, source selection, data charting, synthesis, and characteristics of the included evidence (Haddaway et al., 2022; Page et al., 2021; Paré et al., 2015). Database searches were conducted between 13 March and 17 August 2025. The search strategy was developed to ensure broad coverage of the relevant literature. Section 2.2 describes the general search strategy and its adaptation to each database, including the fields, Boolean combinations, limits, and search dates.

The protocol for this scoping review was retrospectively registered on the Open Science Framework (OSF) and is publicly available at: https://osf.io/5xmyj/ (accessed on 17 August 2025). PRISMA-ScR provides the reporting architecture documenting how the evidence base was constructed, while critical interpretive synthesis provides the analytical logic through which heterogeneous evidence was compared and theoretically integrated. These functions are complementary: evidence mapping establishes methodological traceability, while interpretive synthesis supports explanation and theory development.

Methodological rigor was pursued at two interconnected levels. At the review level, predefined eligibility criteria, structured database searching, documented screening and selection, systematic data charting, and transparent corpus reporting strengthened reproducibility. At the analytical level, rigor was supported through iterative coding, cross-study comparison, reflexive interpretation, conceptual saturation, external feedback, and explicit differentiation between source-level evidence and higher-order interpretations. This approach follows broader methodological guidance emphasizing alignment among review purpose, corpus construction, analytical procedures, and theoretical contribution (Templier & Paré, 2015).

Critical interpretive synthesis was used because the study extends beyond describing the distribution of evidence. The analysis sought recurrent explanatory mechanisms and boundary conditions across heterogeneous sources while retaining contextual sensitivity. Conceptual saturation was understood as the point at which further interpretive comparison no longer generated substantively new analytical categories or explanatory relationships relevant to the review objectives. Importantly, saturation informed the analytical stage rather than the predefined eligibility and selection procedures used to construct the scoping review corpus (Greenhalgh et al., 2018).

Leadership was therefore examined as a context-dependent and emotionally embedded organizational process rather than as fixed categories with universally stable effects. The synthesis focused on interactions among leadership orientations, emotional regulation, contextual pressure, psychological safety, trust, psychological capital, and employee mental and emotional health. This positioning provided the basis for identifying recurrent relationships and subsequently developing the three analytical propositions examined in the results and discussion.

### 2.2. Search Strategy and Eligibility Criteria

A structured literature search was conducted from 13 March to 17 August 2025, across Web of Science Core Collection, Scopus, PubMed, and PsycINFO. These databases were selected to provide complementary coverage of organizational psychology, leadership and management studies, behavioral sciences, occupational health, and mental health research. The search focused on sources published between 2020 and 2025. This temporal boundary was selected to capture leadership and employee mental health during a period characterized by the COVID-19 pandemic and subsequent organizational disruptions, including accelerated digitalization, expansion of hybrid work, labor transformation, geopolitical instability, and sustained uncertainty. Earlier methodological and foundational theoretical references were used exclusively to support the design and conceptual positioning of the review and were not considered part of the 43-source review corpus.

The search strategy combined three concept domains: leadership, disruption or contextual pressure, and mental, emotional, or psychosocial health. The overarching Boolean structure was:

(“leadership” OR “leadership styles” OR “transformational leadership” OR “ethical leadership” OR “servant leadership” OR “inclusive leadership” OR “authoritarian leadership” OR “toxic leadership” OR “destructive leadership” OR “laissez-faire leadership” OR “health-oriented leadership”) AND (“crisis” OR “COVID-19” OR “pandemic” OR “uncertainty” OR “systemic disruption” OR “extreme contexts” OR “organizational disruption”) AND (“mental health” OR “emotional well-being” OR “occupational health” OR “burnout” OR “emotional exhaustion” OR “psychological distress” OR “resilience” OR “psychological safety” OR “psychological capital”).

This overarching strategy was translated into the syntax and indexing structure of each database while preserving the same three conceptual domains and Boolean logic. In Web of Science Core Collection, the terms were searched through the Topic (TS) field; in Scopus, equivalent combinations were applied to TITLE-ABS-KEY; in PubMed, free-text terms were combined with relevant MeSH-related descriptors where appropriate; and in PsycINFO, equivalent free-text terms were combined with corresponding APA Thesaurus-related terminology. Searches were restricted to the 2020–2025 publication period and were conducted within the 13 March–17 August 2025, search window. The final search update across the four databases was completed on 17 August 2025.

Complementary targeted searches were conducted within the same databases to increase sensitivity to potentially relevant leadership–health relationships. These included narrower combinations such as “COVID-19 AND leadership,” “leadership AND burnout,” “crisis leadership AND mental health,” “leadership AND psychological safety,” and “toxic leadership AND burnout.” These targeted searches supplemented the overarching strategy when relevant relationships were expressed through more specific terminology. The combination of the reported Boolean structure, database-specific search fields and controlled vocabulary, publication limits, targeted searches, and search dates provides the information necessary to document and reproduce the search procedure. Search results were exported to Zotero for reference management, duplicate identification and removal, organization of records, and documentation of the subsequent screening process.

Eligibility criteria were defined before final corpus construction and applied consistently throughout screening. Sources were eligible when they met the following criteria: (1) peer-reviewed scholarly publications, including empirical journal articles and conceptual, review-based, or theoretical contributions; (2) publication between 2020 and 2025; (3) explicit examination of leadership within organizational, healthcare, educational, military, public-sector, hospitality, or service settings; (4) consideration of at least one mental, emotional, or psychosocial construct relevant to employee health, including well-being, burnout, emotional exhaustion, psychological distress, resilience, psychological safety, trust, organizational commitment, psychological capital, or closely related outcomes; and (5) substantive relevance to understanding how leadership operates under disruption, uncertainty, crisis, or sustained organizational pressure.

Sources were excluded when they were editorials, non-scholarly reports, conference abstracts, or publications without sufficient theoretical or methodological information for meaningful interpretation. Studies addressing leadership exclusively in relation to competencies, training, performance, or organizational outcomes without a discernible connection to employee mental, emotional, or psychosocial health were also excluded. Duplicate records and sources for which sufficient information could not be retrieved for eligibility assessment were removed from consideration. Studies whose connection to the review concepts and context remained peripheral after full-text assessment were excluded at the eligibility stage.

The eligibility framework was intentionally broad enough to accommodate methodological heterogeneity while sufficiently bounded to preserve conceptual coherence. Empirical and conceptual sources were therefore eligible for different but complementary purposes: empirical studies provided evidence concerning relationships among leadership, psychosocial mechanisms, and employee outcomes, whereas conceptual and review-based contributions supported the mapping, comparison, and interpretation of theoretical explanations across the field. This distinction was retained during data charting and synthesis rather than collapsing heterogeneous forms of evidence into a single evidentiary category.

### 2.3. Study Selection and Corpus Description

The initial database search yielded 428 records. After duplicate removal in Zotero, 391 unique records remained. One reviewer conducted the primary title-and-abstract screening and subsequent full-text eligibility assessment against the predefined eligibility criteria to identify relevant sources addressing leadership and employee mental or emotional health under crisis, disruption, uncertainty, or sustained organizational pressure. The same predefined criteria were applied at both screening stages. Eligibility was determined by substantive alignment with the review concepts and context, while conceptual clarity, contextual relevance, and methodological transparency informed subsequent data charting and interpretive synthesis.

The final corpus comprised 43 sources: 30 empirical studies and 13 conceptual, review-based, or theoretical contributions. These sources constitute the evidence base of the scoping review and reflect the methodological and conceptual heterogeneity of the field. The corpus was intended to map and integrate the range of available evidence rather than provide a statistically representative sample or support effect-size estimation or statistical generalization.

Corpus construction was determined by the predefined eligibility criteria rather than by analytical saturation. Conceptual sufficiency and saturation were considered during the subsequent interpretive synthesis. Conceptual sufficiency was observed when the included evidence provided substantive coverage of the principal leadership orientations, psychosocial mechanisms, and mental health outcomes relevant to the review objective. Analytical saturation was identified when iterative comparison of the included sources no longer generated substantively new explanatory mechanisms or conceptual relationships. Three higher-order analytical domains consequently emerged: prosocial leadership and emotional containment; dysfunctional leadership and emotional dysregulation under pressure; and psychosocial mechanisms linking leadership with employee mental and emotional health.

The selection and analytical refinement process incorporated reflexive feedback from two academic collaborators with expertise in leadership and organizational research. Although they did not perform independent duplicate screening, they provided external critical feedback on borderline eligibility decisions, conceptual coherence, and the consistency of emerging analytical categories. Accordingly, their contribution functioned as reflexive validation rather than as a substitute for independent dual screening. This procedure strengthened interpretive rigor while maintaining explicit transparency about the single-reviewer selection process.

**Figure 1:**
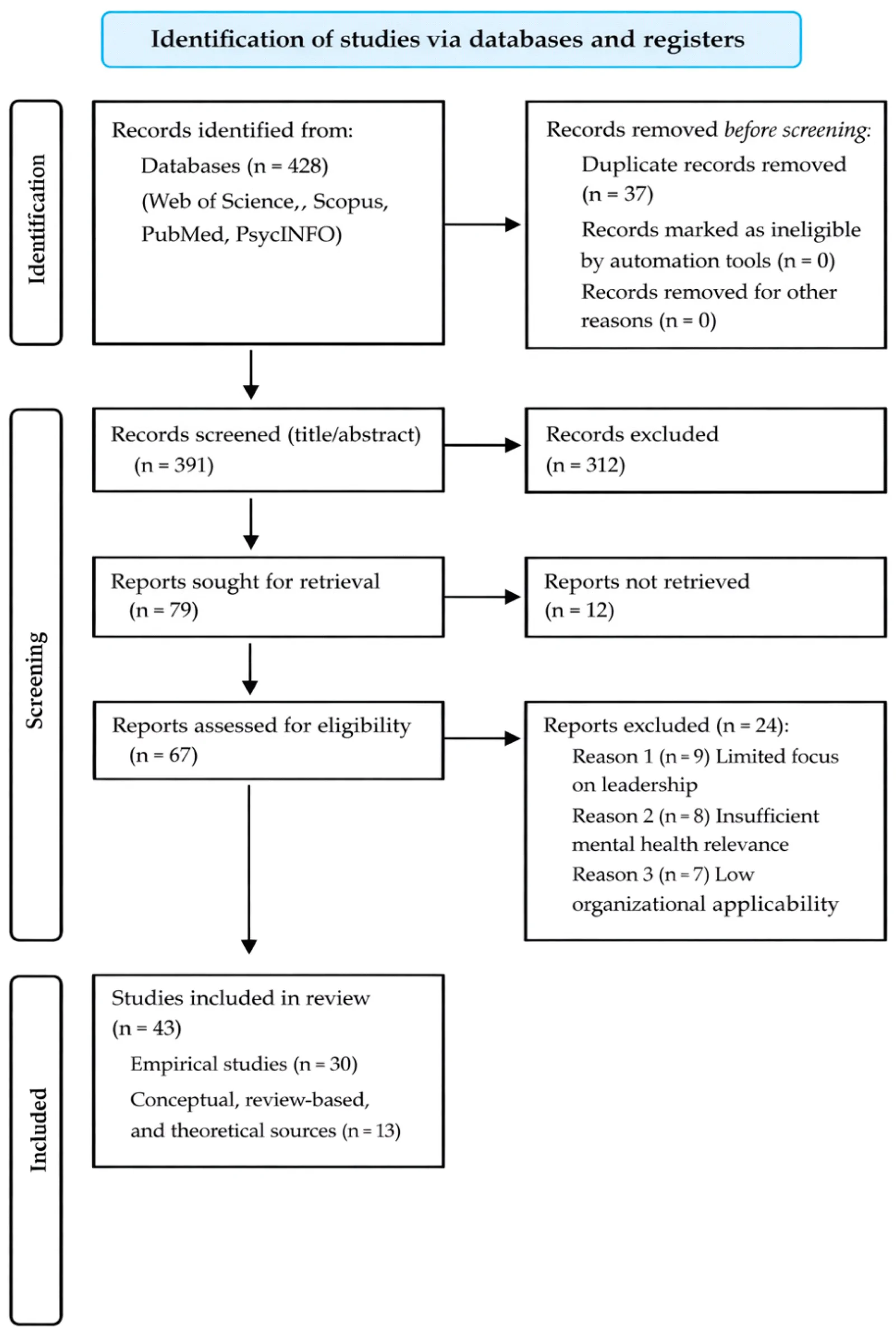
PRISMA-ScR flow diagram of the source selection process.

Figure 1 presents the identification, duplicate removal, title and abstract screening, full-text eligibility assessment, exclusions, and final inclusion of sources using a PRISMA-ScR-aligned flow structure. The diagram provides a transparent account of corpus construction and complements the detailed eligibility and selection procedures reported above.

### 2.4. Data Charting, Analytical Synthesis, and Quality Considerations

The analytical process combined structured data charting, iterative coding, constant comparison, and critical interpretive synthesis. In the first analytical cycle, information from each source was charted according to leadership approach, organizational context, type of disruption or pressure, mental and emotional health outcomes, mediating or moderating variables, methodological design, and broader organizational consequences. These data were organized in a comparative matrix that enabled systematic cross-source analysis while preserving the contextual and methodological characteristics of each contribution.

In the second cycle, repeated reading and cross-source comparison were used to identify convergences, divergences, contradictions, and explanatory gaps. Particular attention was given to psychosocial mechanisms linking leadership with mental and emotional health outcomes, including psychological safety, trust, psychological capital, ethical climate, organizational justice, identity resources, rumination, stress, and anxiety-related processes. Categories were iteratively refined according to their explanatory relevance and consistency across the evidence rather than descriptive similarity alone.

In the third cycle, the analysis moved from evidence mapping toward higher-order interpretive synthesis. Three analytical domains emerged: prosocial leadership and emotional containment; dysfunctional leadership and emotional dysregulation under pressure; and psychosocial mechanisms linking leadership with employee mental and emotional health. These domains provided the analytical structure for the results and informed the subsequent development of P1, P2, and P3. Throughout this stage, relationships directly reported or recurrently supported across included sources were distinguished from higher-order explanatory relationships generated through synthesis. The latter were treated as theoretically plausible propositions requiring subsequent empirical testing rather than as effects established by the reviewed evidence.

Given the methodological heterogeneity of the corpus, quality considerations were incorporated to inform interpretation rather than to establish rigid exclusion thresholds. Sources were considered according to four dimensions: conceptual clarity, methodological transparency, empirical or theoretical contribution, and contextual relevance. Evidence demonstrating stronger conceptual articulation, methodological consistency, and alignment with the review objective received greater interpretive weight, whereas more narrowly focused contributions were integrated cautiously. This emphasis is consistent with critical reflections on review-based research, which caution that purely descriptive mapping may obscure the theoretical significance of heterogeneous evidence (Schlütter et al., 2024).

Appraisal was differentiated according to source type. Empirical studies were considered in relation to design coherence, construct clarity, sample relevance, and correspondence between reported findings and conclusions. Conceptual and review-based sources were considered according to theoretical clarity, contribution to synthesis, and explanatory relevance to leadership–health relationships. This differentiated approach avoided imposing a single evidence hierarchy on sources serving distinct epistemological functions.

Several limitations and potential sources of bias were considered when interpreting the evidence. Many empirical studies relied on cross-sectional and self-report designs, restricting causal inference. Healthcare, education, hospitality, and service settings were particularly prominent, while Western and Asian contexts were more strongly represented than Global South settings. Publication bias may have increased the visibility of statistically significant leadership–health relationships. Interpretive bias was managed through explicit analytical criteria, documented selection, iterative coding, external reflexive feedback, and systematic separation of source-supported relationships from synthesis-generated propositions.

The review was conducted and reported as a scoping review in accordance with PRISMA-ScR. Critical appraisal was used to contextualize and interpret heterogeneous evidence rather than as a mandatory eligibility criterion or basis for quantitative evidence grading. Accordingly, statistical procedures associated with meta-analysis, including pooled effect estimation, statistical heterogeneity assessment, and sensitivity analysis, were outside the scope and objectives of this review.

## 3. Results

### 3.1. Characteristics of the Included Studies

The final corpus included 43 sources published between 2020 and 2025: 30 empirical studies and 13 conceptual, review-based, or theoretical contributions. This composition reflects the integrative logic of the review. Empirical studies provided evidence on leadership styles, psychosocial mechanisms, and employee health outcomes, whereas conceptual and review-based sources clarified crisis leadership, healthy leadership, destructive leadership, servant leadership, methodological transparency, and theoretical integration.

The empirical corpus was methodologically diverse but unevenly distributed. Most studies relied on quantitative cross-sectional designs, frequently using mediation, moderation, or structural equation modeling. These designs were especially visible in studies examining transformational leadership and well-being, ethical leadership, servant leadership, inclusive leadership, and dysfunctional leadership. A smaller but analytically important subset included longitudinal, intervention, qualitative, validation, and mixed-method approaches. This distinction matters because the cross-sectional evidence supports associations between leadership and mental health, whereas longitudinal and intervention studies provide stronger support for interpreting leadership as a process unfolding over time. Linvill and Onosu (2023) qualitative analysis of empathic leadership during the COVID-19 pandemic is particularly relevant because it captures the lived emotional meaning of leadership beyond variable-based models. Similarly, Ertem (2024) mixed-method contribution broadens the corpus by showing how school leadership and mental health intersect in educational crisis contexts.

The corpus also shows sectoral and geographical variation. Healthcare, hospitality, education, public organizations, military contexts, and general employee samples are represented. Asian and European settings are especially visible, while North American studies appear through research conducted in the United States and Canada. African and Global South perspectives remain less frequent, although Ibero-American contributions add contextual sensitivity regarding ethical leadership, psychological safety, occupational mental health, and organizational climate. This distribution suggests that the evidence base is sufficiently diverse for interpretive synthesis, but not geographically balanced enough to support universal claims. Table 1 summarizes the descriptive characteristics of the included sources.

### 3.2. P1: Prosocial Leadership, Emotional Regulation, and Conditional Protection

The first analytical pattern concerns the generally protective associations of prosocial leadership and the conditions that may shape them. Across the corpus, transformational, ethical, servant, inclusive, and health-oriented leadership are generally associated with psychological safety, trust, resilience, engagement, commitment, job satisfaction, psychological capital, and well-being (Tafvelin et al., 2025). However, the synthesis does not support the assumption that these styles are automatically protective. Rather, it suggests that their protective potential may vary with the emotional and relational functions performed under pressure and with leaders’ capacity to sustain them consistently.

Transformational leadership illustrates this interpretive possibility. In hospitality, organizational, military, and crisis-related contexts, it is associated with psychological well-being, engagement, job satisfaction, work performance, and psychological capital (Abolnasser et al., 2023; Khan et al., 2020; Kloutsiniotis et al., 2022; Lester et al., 2024; Njaramba, 2024). Yet its protective value does not seem to derive simply from inspiration or vision. Rather, the reviewed evidence suggests benefits when leaders help employees reinterpret uncertainty, maintain purpose, and preserve psychological resources. The synthesis therefore raises the possibility that its protective potential may vary across contexts, particularly when meaning-making and emotional availability become difficult to sustain under pressure.

Servant leadership follows a different protective pathway. Its value lies less in inspirational alignment and more in relational care, trust, psychological safety, identity, and resilience. Ahmed et al. (2023) show that servant leadership reduces burnout through psychological safety and trust, while Peng et al. (2023) link servant leadership with employee resilience through social identity processes. Zada et al. (2022) suggest that servant leadership can reduce psychological distress in crisis contexts through engagement and mindfulness-related mechanisms, and Schowalter and Volmer (2024) connect it with adaptivity and proactivity. These studies indicate that care-oriented leadership is associated with favorable outcomes when relational support is converted into usable psychological resources. Irving (2025) further qualifies this interpretation by showing that servant leadership must be culturally situated rather than treated as a universally identical construct across contexts.

Ethical leadership introduces the clearest ambivalence within the prosocial group. It is associated with organizational justice, moral identity, affective commitment, and reduced exhaustion when it provides clarity and fairness (Alhaidan, 2025; Santiago-Torner et al., 2024b). Crisis-oriented contributions also emphasize authentic and ethical leadership as relevant during recovery and uncertainty (Keselman & Saxe-Braithwaite, 2021; Markey et al., 2021). However, Wang and Hannah (2025) show that organizational ethical pressure may threaten employee health when moral demands become excessive or unsupported, even though ethical leadership and ethical efficacy can buffer these effects. This suggests that the protective potential of ethical leadership may depend on providing moral clarity without producing moral overload (Santiago-Torner et al., 2025).

Inclusive and health-oriented leadership reinforce the relevance of contextual and resource conditions. Inclusive leadership can reduce emotional exhaustion through caring ethical climate and psychological safety (X. Li & Peng, 2022). Health-oriented leadership functions as a job resource and may buffer job demands when leaders can sustain staff care, health awareness, and supportive behaviors over time (Klebe et al., 2021; Krick et al., 2022). Leadership interventions may also improve health-related outcomes when they strengthen supervisor skills and mindfulness-based capacities (Vonderlin et al., 2023). Together, these findings support the relevance of leader and organizational resources, although they do not directly establish emotional regulation as a moderator of prosocial leadership effects.

Taken together, the reviewed evidence supports recurrent associations between prosocial leadership and favorable employee outcomes but does not directly test the moderation proposed in P1. Building on these associations, the interpretive synthesis generates P1 as a theoretically plausible boundary condition: leaders’ emotional regulation capacity and psychological resources may shape whether the protective potential of prosocial leadership can be sustained under disruption. P1 therefore represents a synthesis-generated proposition requiring direct empirical testing rather than an established moderation effect.

### 3.3. P2: Dysfunctional Leadership, Crisis Pressure, and Emotional Dysregulation

The second analytical pattern concerns dysfunctional leadership. The corpus provides consistent evidence that authoritarian, toxic, destructive, and laissez-faire leadership are associated with harmful psychosocial outcomes, including emotional exhaustion, burnout, psychological distress, rumination, silence, turnover intentions, reduced helping behavior, weakened organizational climate, and depletion of psychological capital. However, the central analytical contribution is not simply that dysfunctional leadership is harmful. Rather, the synthesis raises the possibility that contextual pressure and reduced leader resources may contribute to the persistence or intensification of harmful leadership behaviors during crises.

Authoritarian leadership shows how the search for control may become psychologically costly. Asim et al. (2021) show that authoritarian leadership affects employee helping behavior through rumination, while Zhang et al. (2022) connect authoritarian leadership with cyberloafing through emotional exhaustion and power distance orientation. Zheng et al. (2025) show that authoritarian leadership contributes to young nurses’ burnout through organizational climate and psychological capital. These studies suggest that authoritarian leadership is associated with reduced autonomy, constrained voice, cognitive–emotional rumination, and depleted psychosocial resources. Under crisis pressure, control-oriented behaviors may appear functional because they promise speed and order. Yet the evidence indicates that they often increase strain and reduce adaptive capacity.

Toxic and destructive leadership present a stronger and more consistent pattern of damage. Farghaly Abdelaliem and Abou Zeid (2023) link toxic leadership with nurses’ silence and poorer organizational performance. Nunes and Palma-Moreira (2024) connect toxic leadership with turnover intentions through burnout. Palvimo et al. (2023) associate destructive leadership with burnout among nurses, while P. Li et al. (2024) provide longitudinal meta-analytic evidence showing that destructive leadership has negative consequences for employee outcomes over time. Labrague (2024) further demonstrates that toxic leadership affects nursing workforce outcomes and patient safety. These findings show that destructive leadership is associated not only with poorer individual well-being but also with weakened voice, reliability, service quality, and organizational learning.

Laissez-faire leadership is less visibly aggressive but remains harmful because it withdraws guidance, structure, and emotional support when employees most need clarity. In crisis conditions, passive leadership may be experienced as emotional abandonment. Aslan et al. (2025) show that leadership styles influence work stress through workplace climate and feelings of entrapment. This suggests that the absence of leadership can increase uncertainty, reduce psychological safety, and intensify employees’ perception of being trapped in uncontrollable conditions.

The conceptual literature helps explain why dysfunctional leadership should not be attributed exclusively to stable destructive traits. Crisis leadership research emphasizes that leadership behavior is shaped by sense-making demands, relational practices, contextual intensity, and emotional strain (Lehtonen et al., 2025; Riggio & Newstead, 2023; Wu et al., 2021). Building on this literature, the synthesis proposes that prolonged disruption may deplete leaders’ psychological resources, reduce tolerance for ambiguity, and challenge emotional regulation. These conditions may plausibly contribute to authoritarian control, avoidance, irritability, moral disengagement, or neglectful behavior.

The reviewed evidence therefore establishes harmful associations between dysfunctional leadership and employee outcomes but does not directly demonstrate the crisis-driven escalation mechanism proposed in P2. The interpretive synthesis generates P2 as a theoretically plausible explanation whereby organizational pressure, resource scarcity, leader burnout, emotional dysregulation, and instability may reduce leaders’ capacity to respond constructively. This interpretation does not excuse harmful leadership. It expands the explanatory model by connecting destructive leadership with psychosocial risk systems. Contextual pressures may also privilege speed, compliance, control, and short-term output over emotional sustainability. Prevention therefore requires organizational-level attention to how performance pressures, accountability systems, and resource constraints may shape leadership behavior.

### 3.4. P3: Psychosocial Mechanisms Linking Leadership with Mental and Emotional Health

The third analytical pattern concerns the mechanisms through which leadership relates to mental and emotional health. Across the corpus, psychological safety, trust, psychological capital, ethical climate, organizational justice, identity resources, rumination, stress, and anxiety repeatedly appear as mediators, moderators, or explanatory variables. The central finding is that these mechanisms should not be treated as isolated variables. Rather, the synthesis suggests that they may operate as interconnected psychosocial processes through which leadership is associated with emotional exhaustion and resilience.

Psychological safety appears as a prominent layer of emotional protection. Servant, inclusive, and transformational leadership are especially relevant in this regard. Ahmed et al. (2023) show that psychological safety mediates the relationship between servant leadership and burnout, with trust in the leader further strengthening this protective pathway. X. Li and Peng (2022) show that caring ethical climate and psychological safety explain how inclusive leadership reduces emotional exhaustion. These findings suggest that psychological safety reduces perceived threat and creates relational conditions in which employees can express concerns, seek support, and engage with uncertainty without excessive fear.

Trust represents another central mechanism. Ethical, servant, and authentic leadership generate trust by making leaders’ intentions more predictable and by stabilizing relational expectations (Ahmed et al., 2023; Alhaidan, 2025; Keselman & Saxe-Braithwaite, 2021; Markey et al., 2021; Plouffe et al., 2023). In crisis contexts, trust matters because employees face intensified ambiguity and heightened vulnerability. When trust is present, organizational demands are more likely to be interpreted as legitimate and manageable. When trust is absent, the same demands may be experienced as arbitrary, threatening, or exploitative.

Psychological capital and resilience constitute a further mechanism. Transformational leadership and psychological capital are linked with health outcomes in extreme and crisis contexts (Lester et al., 2024; Njaramba, 2024). Servant leadership contributes to resilience and psychological protection through social identity and engagement-related mechanisms (Peng et al., 2023; Zada et al., 2022). Conversely, authoritarian leadership may reduce psychological capital, thereby increasing burnout (Zheng et al., 2025). These findings suggest that psychological capital is not simply an individual resource independent of leadership. It is partly shaped by the relational and emotional conditions leaders create.

Burnout and emotional exhaustion require conceptual precision because they function as central outcomes across the corpus. Although several studies examine burnout as a consequence of harmful leadership or as an outcome reduced by supportive leadership, measurement-oriented contributions remain important for interpreting these relationships consistently. Mazzetti et al. (2022) strengthen the occupational health relevance of the synthesis by providing validation evidence for the BAT-12, thereby supporting a more precise assessment of burnout in organizational research.

Other mechanisms extend this interconnected pattern. Organizational justice, ethical climate, and occupational mental health strengthen commitment and reduce strain (Alhaidan, 2025). Rumination, worry, stress, and anxiety processes explain how harmful leadership becomes internalized as cognitive–emotional burden (Asim et al., 2021; Kloutsiniotis et al., 2022; Zhang et al., 2022). Empathy shows an ambivalent role, especially in helping professions, where emotional attunement can support care but also contribute to burnout when not accompanied by boundaries and resources (Stosic et al., 2022).

The reviewed evidence supports the relevance of psychological safety, trust, and psychological capital as interconnected mechanisms, but does not establish a fixed temporal sequence among them. P3 therefore represents a synthesis-generated proposition in which psychological safety, trust, and psychological capital may interact in shaping emotional exhaustion and resilience. The proposed sequence among these constructs should be understood as a theoretically plausible pathway requiring direct empirical testing rather than as a demonstrated causal process. This interpretation integrates previously fragmented mechanisms into a coherent framework and offers a stronger theoretical bridge between leadership research, organizational psychology, and occupational health. It also suggests that leadership may influence health not only through stress-related processes, but also by shaping the psychosocial resources employees use to cope with them.

### 3.5. Integrated Thematic Synthesis

The thematic distribution of the corpus reinforces the three-propositions structure. Transformational, servant, ethical, health-oriented, authoritarian, toxic/destructive, and crisis/relational leadership are the most visible categories. Burnout and emotional exhaustion are the most frequent outcomes, followed by psychological distress, psychological well-being, resilience, job satisfaction, engagement, and commitment. Mechanisms such as organizational climate, ethical climate, psychological capital, psychological safety, trust, rumination, stress, and anxiety appear repeatedly, although they are often examined separately.

Table 2 shows that post-2020 literature has concentrated strongly on leadership styles traditionally viewed as protective, especially transformational, servant, ethical, and health-oriented leadership. However, frequency should not be confused with unconditional effectiveness. The synthesis therefore proposes that the protective potential of these styles may vary with leaders’ capacity to regulate emotional demands and sustain supportive behavior under pressure. Conversely, dysfunctional leadership styles are less frequent in the corpus but produce more consistently detrimental outcomes. This asymmetry supports the need to study both protective and harmful leadership through the lens of emotional regulation and psychosocial resources.

**Figure 2:**
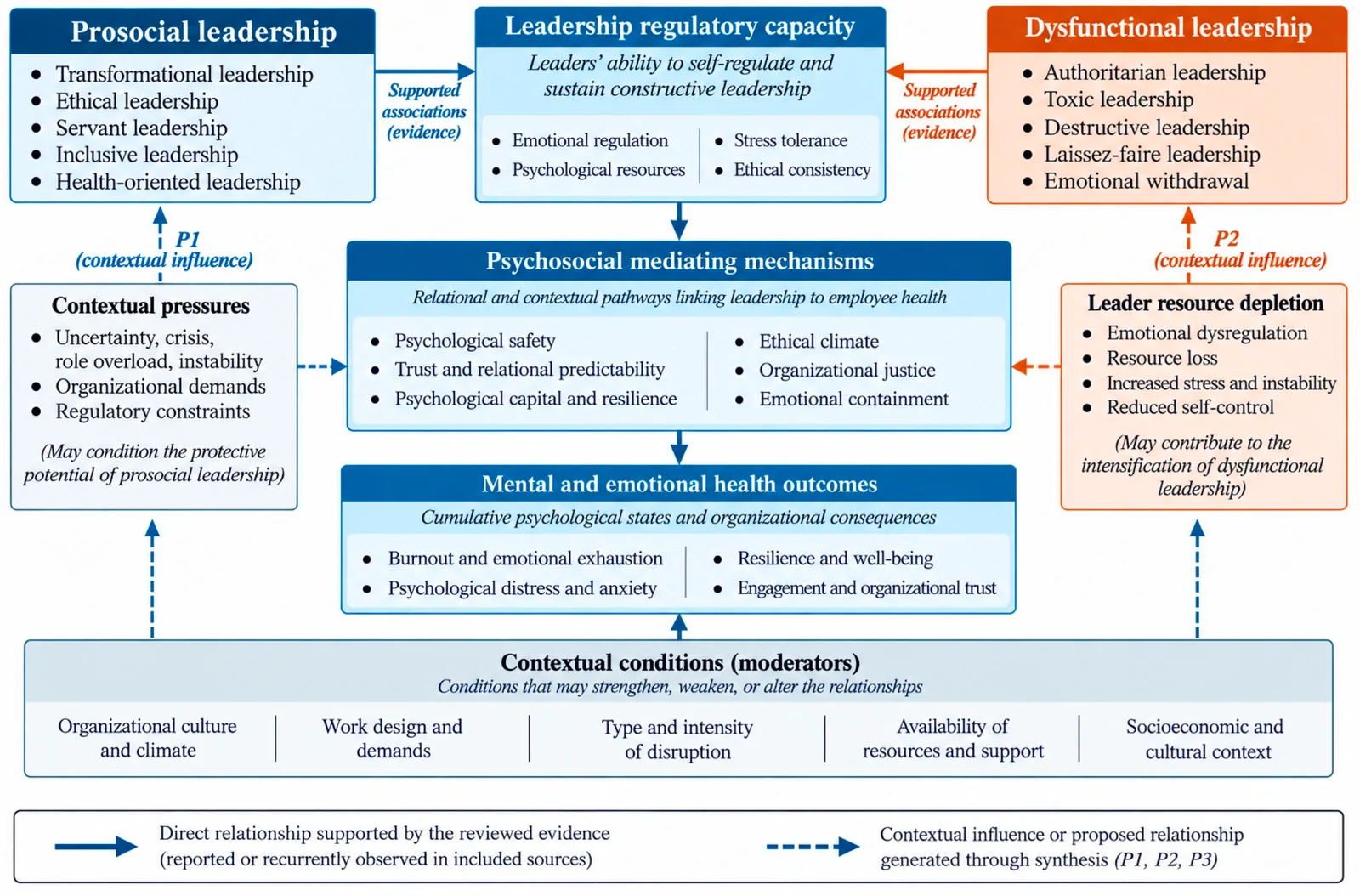
Dynamic emotional regulation framework of leadership under disruption: evidence-supported relationships and synthesis-generated propositions. Note. Solid arrows indicate relationships directly supported by the reviewed evidence. Dashed arrows indicate contextual influences or theoretical relationships generated through the critical interpretive synthesis and requiring empirical testing (P1, P2, and P3). The framework is interpretive and should not be understood as a validated causal model.

Figure 2 translates the synthesis into a conceptual model. The figure represents leadership not as a set of isolated styles, but as a dynamic process that connects leadership orientation, leaders’ emotional regulation capacity, psychosocial mechanisms, and employee outcomes. Importantly, the model distinguishes relationships directly supported by the reviewed evidence from contextual influences and higher-order relationships generated through the critical interpretive synthesis. Prosocial leadership is associated with favorable outcomes, while P1 proposes that its protective potential may vary with leaders’ regulatory resources. Dysfunctional leadership is associated with adverse outcomes, while P2 proposes that crisis-related resource depletion and dysregulation may contribute to its intensification. P3 integrates psychological safety, trust, and psychological capital as interconnected mechanisms whose proposed relationships require direct empirical testing.

Overall, the results support moving beyond style-based description toward process-based explanation. The reviewed evidence identifies recurrent associations between leadership and mental and emotional health, while the synthesis proposes that these relationships may operate through regulatory, relational, and contextual processes. Under pressure, leadership may be associated with the preservation or erosion of psychological safety, trust, psychological capital, and employee well-being. These relationships, together with the synthesis-generated propositions represented by the dashed pathways, provide the basis for the discussion and for the proposed theoretical and practical contributions.

The integrated synthesis also highlights a temporal proposition. Mental and emotional health outcomes may develop through repeated interactions, perceived patterns, and employees’ interpretation of whether leaders are predictable, fair, emotionally available, and capable of managing pressure. Because the predominantly cross-sectional evidence does not establish this temporal ordering, longitudinal research is needed to examine how these relationships evolve over time. This temporal perspective therefore represents an additional direction for empirical examination of leadership as a continuing relational process rather than as a one-time predictor of employee outcomes.

## 4. Discussion

This scoping review with critical interpretive synthesis addresses a central gap in the leadership and occupational health literature. Although a substantial body of research has associated leadership styles with employee well-being, burnout, resilience, psychological safety, and organizational adaptation, considerably less attention has been devoted to integrating the dynamic psychosocial processes through which leadership may protect or undermine mental and emotional health under disruption. The synthesis suggests that this gap cannot be adequately addressed through a simple distinction between “positive” and “negative” leadership styles. Instead, leadership emerges as a relational and context-dependent process involving emotional regulation, psychosocial resource preservation, and collective sense-making under conditions of uncertainty and sustained pressure.

The reviewed evidence indicates that prosocial leadership styles are generally associated with more favorable employee outcomes. Transformational, servant, ethical, inclusive, and health-oriented leadership have been linked to psychological well-being, engagement, resilience, trust, psychological capital, and lower emotional exhaustion (Ahmed et al., 2023; Khan et al., 2020; Klebe et al., 2021; Kloutsiniotis et al., 2022; Krick et al., 2022; X. Li & Peng, 2022; Njaramba, 2024; Peng et al., 2023; Zada et al., 2022). Importantly, however, the reviewed studies do not directly establish emotional regulation as a moderator of these associations. Building on them, P1 proposes that leaders’ regulatory capacity may constitute a boundary condition shaping whether the protective potential of prosocial leadership can be sustained under disruption.

This possibility can be considered across different prosocial leadership approaches. Transformational leadership appears beneficial when it enables employees to interpret uncertainty, preserve meaning, and maintain psychological resources in demanding environments (Khan et al., 2020; Lester et al., 2024; Njaramba, 2024). Servant leadership contributes to well-being when it strengthens trust, psychological safety, identity, and resilience (Ahmed et al., 2023; Peng et al., 2023; Schowalter & Volmer, 2024; Zada et al., 2022). Similarly, health-oriented leadership functions as a protective resource when leaders recognize strain, facilitate recovery, and sustain health-promoting behaviors over time (Klebe et al., 2021; Krick et al., 2022; Vonderlin et al., 2023). Inclusive leadership is also relevant because of its association with reduced emotional exhaustion through caring ethical climates and psychological safety (X. Li & Peng, 2022). Taken together, these findings suggest that the favorable associations of prosocial leadership may reflect specific practices providing emotional containment, relational predictability, and continued access to psychosocial resources.

Such an interpretation also prevents an overly idealized understanding of positive leadership. Ethical leadership, for example, is commonly associated with trust, justice, moral clarity, and responsible conduct (Alhaidan, 2025; Markey et al., 2021). Yet the evidence reviewed also points to a more complex relationship between ethical expectations and employee health. Santiago-Torner et al. (2024b) connect ethical leadership with emotional exhaustion and affective commitment through moral and affective processes, while Wang and Hannah (2025) show that organizational ethical pressure may generate strain when moral demands exceed available resources. Ethical leadership may therefore be most protective when moral clarity is accompanied by feasible expectations, organizational fairness, adequate resources, and emotional sustainability. This reinforces the broader argument that the health implications of leadership cannot be inferred from leadership labels alone; they may vary according to how leadership is experienced within particular relational and organizational conditions.

A contrasting pattern emerges for dysfunctional leadership. Authoritarian, toxic, destructive, and laissez-faire leadership are repeatedly associated with burnout, psychological distress, organizational silence, cynicism, turnover intentions, and deteriorated organizational climates (Asim et al., 2021; Farghaly Abdelaliem & Abou Zeid, 2023; Labrague, 2024; P. Li et al., 2024; Nunes & Palma-Moreira, 2024; Palvimo et al., 2023; Zhang et al., 2022; Zheng et al., 2025). Beyond these established associations, however, the synthesis raises a theoretically important possibility: disruptive conditions may contribute to the persistence or intensification of dysfunctional behaviors by depleting leaders’ psychological resources and increasing emotional dysregulation. P2 represents this synthesis-generated escalation mechanism and requires direct empirical testing rather than being interpreted as a causal relationship established by the review.

Authoritarian leadership illustrates elements relevant to this proposed mechanism. Zhang et al. (2022) associate authoritarian leadership with emotional exhaustion through rumination, while Zheng et al. (2025) link authoritarian leadership with burnout through deteriorated organizational climate and reduced psychological capital. Asim et al. (2021) further indicate that authoritarian leadership weakens helping behavior and organizational functioning. Read together, these findings raise the possibility that under uncertainty some leaders may increase control, restrict participation, or suppress ambiguity. Although these responses may temporarily create an appearance of order, they may be accompanied by diminished trust, weakened psychological safety, and reduced employee resilience. The significance of authoritarian leadership therefore lies not only in the behavior itself but also in examining the organizational and emotional conditions under which control may emerge during disruption.

Toxic and destructive leadership follow a related logic, although their consequences appear particularly detrimental. Empirical and review-based evidence associates these forms of leadership with organizational silence, burnout, turnover intentions, reduced trust, and deteriorated work climates (Farghaly Abdelaliem & Abou Zeid, 2023; Labrague, 2024; P. Li et al., 2024; Nunes & Palma-Moreira, 2024; Palvimo et al., 2023). Laissez-faire leadership, despite being less overtly aggressive, may similarly undermine mental health by withdrawing guidance, emotional presence, and decision clarity precisely when employees require greater structure and relational assurance. These patterns are consistent with conceptual approaches that understand crisis leadership as situated, relational, and emotionally demanding rather than simply directive or heroic (Dirani et al., 2020; Lehtonen et al., 2025; Riggio & Newstead, 2023; Wu et al., 2021). The synthesis consequently proposes that dysfunctional leadership might, in some circumstances, reflect organizational strain as well as contribute to psychosocial deterioration. This interpretation does not diminish leader responsibility; rather, it places harmful leadership within the broader system of demands and resources in which leadership behavior develops.

Regarding P3, the reviewed evidence repeatedly identifies psychological safety, trust, and psychological capital as relevant psychosocial mechanisms associated with emotional exhaustion and resilience. Although these constructs are frequently examined as separate mediators, their recurrent presence across the reviewed evidence suggests the possibility of a more integrated process architecture. The synthesis hypothesizes that leadership may influence whether employees experience their environment as psychologically safe; psychological safety may facilitate trust and relational predictability; trust may support the preservation of psychological capital; and psychological capital may subsequently influence resilience and vulnerability to emotional exhaustion. This ordering is not established by the reviewed studies and should be treated as a synthesis-generated pathway requiring direct empirical examination.

Psychological safety constitutes a particularly important mechanism because it enables employees to express concerns, acknowledge vulnerability, seek help, and participate in learning without excessive fear of punishment or exclusion. Ahmed et al. (2023) show that psychological safety mediates the relationship between servant leadership and burnout, while X. Li and Peng (2022) demonstrate that inclusive leadership reduces emotional exhaustion through caring ethical climate and psychological safety. These findings are especially relevant in disrupted organizational environments, where uncertainty can increase employees’ need for interpersonal reassurance and make the perceived consequences of speaking up more salient. Psychological safety can therefore be understood not merely as a desirable workplace condition but as a psychosocial resource through which leadership may influence employees’ capacity to navigate uncertainty.

Trust represents another stabilizing mechanism. Ethical, servant, and transformational leadership contribute to trust by increasing the predictability of leader intentions and reducing interpersonal ambiguity during periods of instability (Ahmed et al., 2023; Alhaidan, 2025; Keselman & Saxe-Braithwaite, 2021; Markey et al., 2021; Plouffe et al., 2023). Trust does not remove the objective demands associated with crisis or disruption; rather, it may alter how those demands are interpreted and experienced. Employees may be better able to tolerate uncertainty when they perceive leaders as fair, competent, transparent, and genuinely concerned with their well-being. In this sense, trust provides a relational bridge between leadership behavior and employees’ capacity to preserve psychological resources under pressure.

Psychological capital constitutes a further mechanism because it reflects employees’ capacity to preserve hope, efficacy, resilience, and optimism under strain. Lester et al. (2024) and Njaramba (2024) connect transformational leadership with psychological capital in demanding contexts, while Peng et al. (2023) and Zada et al. (2022) associate servant leadership with resilience and psychological protection. Conversely, Zheng et al. (2025) show that authoritarian leadership reduces psychological capital and thereby contributes to burnout. Taken together, these findings suggest that psychological capital should not be viewed exclusively as an individual attribute. Its preservation or depletion may also be influenced by leadership behavior and by the organizational climate in which employees confront demanding circumstances.

The combined interpretation of psychological safety, trust, and psychological capital helps clarify why leadership may relate to employee mental and emotional health without assuming a direct or linear relationship with burnout or resilience. Leadership instead contributes to shaping the psychosocial environment within which employees interpret demands, access resources, regulate emotions, and assess whether the organization is sufficiently safe and predictable to sustain effort. This process-oriented interpretation responds directly to the gap identified in the introduction. The literature has generated considerable evidence concerning associations between particular leadership styles and well-being outcomes, but these relationships have often been examined through fragmented mechanisms. Considering these mechanisms jointly offers a more coherent explanation of how leadership–health relationships may unfold under disruption.

The conceptual sources included in the review reinforce the need for such integration. Rudolph et al. (2020) warn that healthy leadership research risks construct proliferation when the mechanisms connecting leadership with health outcomes remain insufficiently clarified. Wong et al. (2025) similarly demonstrate that employee mental health remains a major challenge in service-intensive sectors, while Labrague (2024) shows that the consequences of toxic leadership extend beyond individual distress to workforce stability and patient safety. Crisis leadership scholarship further emphasizes adaptive, relational, and emotionally responsive leadership practices (Dirani et al., 2020; Riggio & Newstead, 2023; Wu et al., 2021), while Lehtonen et al. (2025) conceptualize crisis leadership as a situated practice emerging within specific relational and contextual conditions. Collectively, these perspectives support a move away from isolated leadership constructs toward an explanation centered on processes, resources, relationships, and context.

The 2020–2025 period is particularly informative for developing this interpretation because it exposed the emotional and relational limits of conventional leadership assumptions. The COVID-19 pandemic, hybrid work, accelerated digitalization, labor instability, healthcare strain, and organizational restructuring did more than provide new contexts in which existing leadership theories could be applied. They made visible the extent to which leadership may be experienced as a source of safety, meaning, trust, and emotional orientation under collective pressure. At the same time, these conditions suggest the potential vulnerability of leaders themselves to depletion, ethical strain, and regulatory overload. This dual vulnerability is central to the framework developed through the review: leaders participate in regulating organizational emotional systems while simultaneously remaining embedded within, and affected by, those same systems.

The central contribution of this synthesis is therefore to conceptualize leadership as a form of collective emotional regulation under disruption. Within this framework, prosocial leadership is proposed as potentially protective when associated with psychological safety, trust, and psychological capital; dysfunctional leadership is proposed as potentially more damaging when accompanied by threat, silence, uncertainty, and resource depletion; and their health implications may be shaped by the interaction among leaders’ regulatory capacities, organizational support, contextual demands, and employee psychosocial resources. Rather than treating leadership styles as inherently protective or harmful categories, this perspective emphasizes the processes and conditions through which leadership–health relationships may emerge. In doing so, the review moves beyond static style-based explanations and provides a more relational, contextual, and process-oriented understanding of leadership at the intersection of organizational psychology and occupational health.

### 4.1. Theoretical Contribution

The findings of this review have several theoretical implications. First, leadership theory should move beyond static typologies and consider emotional regulation as a central explanatory dimension. Classical leadership theory has provided a broad foundation for understanding leadership through transformational, ethical, servant, directive, trait-based, and relational approaches (Northouse, 2025). However, the evidence synthesized here suggests that these approaches remain incomplete if they do not explain how leaders regulate uncertainty, contain emotional strain, and preserve psychosocial resources under pressure.

P1 extends this argument by proposing that prosocial leadership may be conditionally protective rather than inherently beneficial. Transformational leadership may inspire employees, but its protective potential may vary with psychological safety and resource preservation (Khan et al., 2020; Lester et al., 2024; Njaramba, 2024). Servant leadership may reduce burnout through trust and psychological safety, while the sustainability of care-oriented behaviors remains theoretically relevant (Ahmed et al., 2023; Peng et al., 2023; Zada et al., 2022). Ethical leadership may promote justice and moral clarity, but it may also become associated with ethical strain if moral expectations exceed available resources (Santiago-Torner et al., 2024b; Wang & Hannah, 2025). Future leadership theory should therefore examine whether emotional regulation capacity functions as a boundary condition for the health-protective potential of prosocial leadership.

Second, dysfunctional leadership should not be conceptualized only as a deviant expression of individual traits. P2 proposes that authoritarian, toxic, destructive, and laissez-faire leadership may also emerge or intensify when crisis conditions reduce leaders’ psychological resources and increase defensive responses. This does not excuse harmful leadership, but offers a testable extension to explanations of its emergence and persistence. Evidence linking dysfunctional leadership with emotional exhaustion, burnout, silence, turnover intentions, and deteriorated climates provides the empirical foundation from which this proposition was generated (Asim et al., 2021; Farghaly Abdelaliem & Abou Zeid, 2023; Labrague, 2024; P. Li et al., 2024; Nunes & Palma-Moreira, 2024; Palvimo et al., 2023; Zhang et al., 2022; Zheng et al., 2025). Complementary literature in educational settings similarly highlights contextual influences on toxic leadership and identifies emotional intelligence and self-regulation as relevant resources for counteracting its harmful manifestations (Anastasiou, 2025). Future research should test whether leader depletion, emotional dysregulation, and contextual strain operate as antecedents or amplifying conditions of destructive leadership.

Third, leadership theory should place psychosocial mechanisms at the center of explanation. Psychological safety, trust, and psychological capital recur across the evidence as relevant mechanisms associated with employee mental and emotional health. P3 integrates these constructs into a hypothesized process architecture rather than corroborating a demonstrated sequence. Psychological safety captures whether employees can express vulnerability; trust reflects whether they can rely on leaders and interpret decisions as legitimate; psychological capital captures their capacity to preserve hope, efficacy, resilience, and optimism under pressure (Ahmed et al., 2023; X. Li & Peng, 2022; Lester et al., 2024; Njaramba, 2024; Peng et al., 2023; Zheng et al., 2025). Future research should examine these mechanisms jointly and test their proposed ordering rather than assuming it.

Fourth, crisis leadership theory should be more explicitly connected with occupational health. Crisis leadership has traditionally emphasized decision-making, communication, sense-making, and adaptation (Dirani et al., 2020; Riggio & Newstead, 2023; Wu et al., 2021). These dimensions remain important, but leadership in disruption may also be evaluated by its capacity to support psychological functioning while enabling adaptation. Lehtonen et al. (2025) reinforce this point by conceptualizing crisis leadership as situated practice, while Rudolph et al. (2020) call for greater theoretical clarity in healthy leadership research.

To situate this framework within established occupational health theory, it is useful to compare it with the Job Demands–Resources (JD-R) model and Conservation of Resources (COR) theory, which have been the dominant explanatory frameworks for burnout and engagement. The JD-R model conceptualizes leadership as a job resource that buffers the health-impairing effects of job demands (Bakker & Demerouti, 2017), while COR theory frames leadership as a contextual resource that helps employees protect and accumulate personal resources (Hobfoll et al., 2018). The framework proposed here does not contradict these models; rather, it offers three testable extensions. First, it proposes emotional regulation capacity of the leader as a potential boundary condition for the resource-providing function of leadership. Second, it hypothesizes an interconnected and potentially sequential relationship among psychological safety, trust, and psychological capital, whose ordering requires empirical examination. Third, it proposes sustained disruption as a contextual condition that may simultaneously affect leaders’ resource availability and employees’ vulnerability, a dynamic requiring longitudinal testing. Thus, the framework complements JD-R and COR by proposing emotional, relational, and temporal specificity at the leadership–health interface.

Overall, this review advances a dynamic emotional regulation framework of leadership under disruption. The framework rests on three synthesis-generated, empirically testable propositions: prosocial leadership may retain greater protective potential when leaders sustain emotional regulation and relational support; dysfunctional leadership may intensify when crisis conditions erode leaders’ psychological resources; and psychological safety, trust, and psychological capital may operate as interconnected mechanisms linking leadership with emotional exhaustion and resilience. This framework moves leadership theory toward a more relational, contextual, and health-centered understanding of organizational life.

### 4.2. Practical Implications

The findings have practical implications for organizations seeking to protect employee mental and emotional health under disruption. A central implication of the review is that leadership effectiveness should not be evaluated exclusively through productivity, strategic execution, or operational efficiency. The synthesis indicates that leadership is associated with the psychosocial conditions under which performance is sustained. Its capacity to create psychological safety, preserve trust, prevent avoidable emotional exhaustion, and maintain psychosocial resources should therefore form part of how organizations define and evaluate effective leadership.

First, organizations should consider emotional regulation capacity in leadership selection, promotion, and development. As proposed in P1, leaders’ emotional regulation may shape the protective potential of prosocial leadership under intensified demands. Selection and promotion systems should therefore assess not only technical expertise, previous performance, or achievement orientation, but also tolerance for uncertainty, reflective capacity, emotional self-regulation, ethical judgment, active listening, and the ability to sustain supportive behavior under pressure. Because this boundary condition remains to be directly tested, such assessment should complement rather than replace established leadership criteria.

Second, leadership development should extend beyond generic emotional intelligence training toward competencies specifically required under disruption. Programs should strengthen crisis-sensitive emotional regulation, psychological safety creation, ethical decision-making under pressure, recovery practices, and early recognition of burnout in both employees and leaders. Such development is particularly relevant in sectors characterized by sustained emotional demands, including healthcare, education, hospitality, public service, and crisis-response settings. Evidence concerning health-oriented leadership and intervention-based leadership development indicates that protective leadership behaviors can be strengthened when organizations provide structured training and appropriate organizational support (Klebe et al., 2021; Krick et al., 2022; Vonderlin et al., 2023). Leadership development should therefore be understood not as an isolated training intervention, but as part of a broader occupational health strategy.

Third, organizations should develop mechanisms for the early detection of deteriorating leadership processes. P2 raises the possibility that, beyond relatively stable individual tendencies, dysfunctional leadership may intensify when leaders experience resource depletion, sustained overload, or emotional dysregulation. Organizations could therefore consider indicators of leadership deterioration alongside conventional measures of employee burnout and turnover. Relevant warning signals may include increasing overcontrol, emotional withdrawal, inconsistent communication, defensive decision-making, declining empathy, avoidance of difficult conversations, recurrent blaming behaviors, and normalization of organizational silence. Early identification may be valuable because the proposed framework suggests reciprocal pressures between deteriorating leadership and team climates.

Fourth, leadership performance systems should incorporate psychosocial indicators alongside operational and financial outcomes. When leaders are rewarded primarily for short-term productivity, target achievement, or financial results, organizations may unintentionally reinforce practices that produce performance at the expense of employee health. Leadership evaluation should therefore consider psychological safety, trust in leadership, perceived fairness, emotional support, burnout and exhaustion, unwanted turnover, organizational silence, and team resilience. Incorporating these indicators would make visible the psychosocial costs that conventional performance metrics may conceal and would help distinguish genuinely sustainable leadership from short-term effectiveness achieved through excessive pressure or resource depletion.

Fifth, organizations should manage psychological safety, trust, and psychological capital as interconnected strategic resources rather than as isolated employee attitudes. P3 proposes that these mechanisms may operate jointly, although their sequencing remains to be empirically established. Interventions should therefore combine opportunities for employees to express concerns without fear with transparent communication, procedural fairness, relational consistency, and practices that strengthen psychological resources. Coaching, mentoring, peer support, reflective learning, team debriefings, and structured opportunities for voice may contribute to this architecture when they are embedded within credible organizational practices. Psychological safety cannot be sustained by encouraging employees to speak if organizational responses subsequently punish vulnerability, just as trust cannot be developed through communication alone when decisions are perceived as inconsistent or unfair.

Finally, organizations should avoid placing the entire emotional responsibility for crisis management on individual leaders. A central implication of the framework developed in this review is that leaders are simultaneously regulators of organizational emotional processes and actors exposed to those same pressures. They may themselves experience overload, moral strain, conflicting demands, and psychological depletion. Sustainable leadership therefore requires organizational infrastructures that preserve leaders’ regulatory resources, including peer support, supervision and reflective spaces, access to psychological resources, distributed decision-making, recovery opportunities, and realistic workload expectations. Supporting leaders should not be understood as protecting them from accountability; rather, it creates conditions in which responsible and health-protective leadership can be sustained.

Taken together, these implications reposition leadership within occupational health strategy. Leadership may function as both a psychosocial protective resource and a psychosocial risk pathway, depending on how leaders regulate pressure, how organizations support them, and how leadership behaviors relate to employees’ access to safety, trust, and psychological resources. Protecting employee mental and emotional health therefore requires more than individual coping or resilience programs. It requires leadership systems capable of sustaining relational predictability, ethical consistency, psychological safety, trust, and recovery while preventing organizational pressure from being translated into avoidable psychological harm.

### 4.3. Limitations and Future Research

This scoping review has several limitations. First, although the review followed predefined eligibility criteria, structured database searches, documented source selection, systematic data charting, and PRISMA-ScR reporting, its critical interpretive component necessarily involves analytical judgment. Primary screening and full-text eligibility assessment were conducted by one reviewer, with two academic collaborators providing reflexive feedback rather than independent duplicate screening. This may have increased susceptibility to selection judgment despite the predefined criteria and external critical review. Accordingly, the breadth of the corpus supports conceptual mapping and theoretical integration, but the findings should not be interpreted as evidence of causal effects or universal relationships across organizational contexts.

Second, important limitations arise from the characteristics of the included evidence. Much of the empirical literature relies on cross-sectional and self-report designs. These studies are valuable for identifying associations, potential mediating mechanisms, and theoretically meaningful patterns, but provide limited evidence regarding temporal ordering and causality. Moreover, the corpus includes both primary studies and review-based contributions, creating potential overlap where reviews synthesize evidence also represented by individual studies. Such sources were interpreted according to their distinct epistemological functions rather than as independent cumulative observations. Longitudinal, multilevel, experimental, diary-based, and experience-sampling designs are therefore needed to examine how leadership behaviors, emotional regulation, psychological safety, trust, psychological capital, and exhaustion interact and evolve over time.

Third, the evidence base shows sectoral and geographical concentration. Healthcare, education, hospitality, and service settings are particularly visible, while Western and Asian contexts are more strongly represented than the Global South. Greater representation of Latin America, Africa, and underrepresented occupational settings is needed to determine whether the relationships identified in this review are context-specific or transferable across cultural, institutional, and organizational environments. Cross-cultural and cross-sector comparisons would be particularly valuable for establishing the boundary conditions of leadership–health relationships.

The three analytical propositions generated through the synthesis provide a focused agenda for future empirical research. P1 should be examined by testing leaders’ emotional regulation capacity as a potential boundary condition of prosocial leadership. Future studies could determine whether transformational, ethical, servant, inclusive, and health-oriented leadership remain protective when leaders experience high workload, moral pressure, emotional exhaustion, or conflicting demands. Longitudinal and diary designs would be particularly useful for identifying fluctuations in regulatory resources and determining when supportive leadership becomes difficult to sustain.

P2 requires examination of the processes through which organizational disruption may contribute to dysfunctional leadership. Future studies should assess whether crisis pressure, leader burnout, resource scarcity, and emotional dysregulation predict authoritarian, toxic, destructive, or laissez-faire behaviors. Such research could distinguish whether dysfunctional leadership predominantly reflects stable individual dispositions, context-induced regulatory failure, situational amplification, or interactions among individual and contextual factors.

P3 warrants direct examination of the interconnected roles of psychological safety, trust, and psychological capital. Although the reviewed evidence repeatedly identifies these mechanisms, they are generally examined separately rather than as an integrated process. Sequential mediation, longitudinal structural models, multilevel analyses, and mixed-method designs could directly test the hypothesized ordering among these constructs and determine how their individual and combined relationships relate to emotional exhaustion and resilience.

Future research should also examine leaders’ own mental health more systematically. Leaders are not merely sources of psychosocial influence but organizational actors whose emotional and psychological resources can deteriorate under sustained pressure. Examining this vulnerability may help test whether leader depletion contributes to the weakening of otherwise supportive leadership or the emergence or intensification of dysfunctional behaviors during disruption.

Greater attention is also required to contextual boundary conditions. Job demands, employment insecurity, national culture, power distance, digital work intensity, resource availability, and organizational justice may alter how leadership behaviors are interpreted and experienced. Directive behavior, for example, may provide clarity in high-risk environments but become psychologically harmful when accompanied by low trust or perceived procedural unfairness. Future leadership–health models should therefore integrate individual, relational, organizational, and contextual levels of analysis.

Finally, qualitative and mixed-method evidence should be expanded. Employees’ and leaders’ narratives can reveal how leadership behaviors acquire emotional meaning, how trust and psychological safety develop or deteriorate, and why silence, exhaustion, or disengagement may emerge even when formal leadership practices appear supportive. Integrating these experiences with longitudinal quantitative evidence would provide a more complete account of the dynamic leadership–health relationships identified through this scoping review.

## 5. Conclusions

This scoping review mapped and critically integrated evidence on how leadership relates to employees’ mental and emotional health in organizational contexts characterized by disruption, uncertainty, and sustained pressure. Across the heterogeneous evidence base, leadership emerges not only as a directive, motivational, or performance-oriented function, but as a relational and emotionally embedded process associated with how employees interpret uncertainty, preserve psychological resources, experience trust and safety, and sustain resilience.

The synthesis generated three interconnected analytical propositions. P1 suggests that leaders’ emotional regulation capacity may constitute a boundary condition shaping the protective potential of prosocial leadership under pressure. P2 proposes that dysfunctional leadership may reflect not only dispositional tendencies but also a possible contribution of resource depletion and emotional dysregulation under disruptive conditions. P3 identifies psychological safety, trust, and psychological capital as interconnected mechanisms whose proposed integration and ordering require direct empirical examination. These propositions should be understood as synthesis-generated, theoretically grounded relationships requiring empirical testing rather than as causal hypotheses confirmed by the review.

The principal theoretical contribution is a dynamic emotional regulation framework of leadership under disruption. The framework moves beyond static classifications of positive and negative leadership styles by integrating leadership orientation, regulatory capacity, psychosocial mechanisms, contextual conditions, and employee health outcomes. It therefore provides a theoretical structure for examining when leadership may contain psychological strain, when its protective capacity may weaken, and when organizational pressure may contribute to harmful leadership processes.

This interpretation also has practical and normative implications. Evaluating leadership primarily through productivity, speed, or formal goal attainment may obscure the psychological costs through which organizational outcomes are achieved. Leadership that produces short-term performance while eroding trust, psychological safety, or employee resources may ultimately undermine adaptation and sustainability. Conversely, protecting mental and emotional health is not peripheral to leadership effectiveness; it contributes to the psychosocial conditions required for learning, engagement, retention, recovery, and responsible organizational functioning.

Leadership under disruption should therefore be evaluated not only by its capacity to mobilize employees through change, but also by its capacity to preserve the psychological conditions that enable people to work, adapt, speak, and recover without avoidable harm. By connecting leadership orientation with regulatory resources, psychosocial mechanisms, and occupational mental health, this review offers a more contextual and process-oriented understanding of leadership relevant to both organizational psychology and occupational health.

## Figures and Tables

**Table 1:** Descriptive characteristics of the included sources (2020–2025).

Ref.	Country	Study	Methodological Design	Sample
Abolnasser et al. (2023)	Egypt	Empirical	Cross-sectional (SEM)	Hotel employees
Ahmed et al. (2023)	China/Pakistan	Empirical	Longitudinal (3-wave panel)	Employees
Alhaidan (2025)	Saudi Arabia	Empirical	Cross-sectional (survey-based)	Employees
Asim et al. (2021)	China	Empirical	Cross-sectional (mediation/moderation)	Employees
Aslan et al. (2025)	Turkey	Empirical	Cross-sectional	Employees
De Clercq and Belausteguigoitia (2021)	Mexico	Empirical	Cross-sectional	Employees
Ertem (2024)	International	Empirical	Mixed-method (secondary data + survey)	School principals
Farghaly Abdelaliem and Abou Zeid (2023)	Egypt	Empirical	Cross-sectional	Nurses
Khan et al. (2020)	Pakistan	Empirical	Cross-sectional (mediation model)	Employees
Klebe et al. (2021)	Germany	Empirical	Longitudinal (panel)	Employees
Kloutsiniotis et al. (2022)	Greece	Empirical	Cross-sectional	Hospitality employees
Krick et al. (2022)	Germany	Empirical	Cross-sectional	Employees
Lester et al. (2024)	USA	Empirical	Field study (quantitative)	Military personnel
X. Li and Peng (2022)	China	Empirical	Cross-sectional (mediation)	Employees
Linvill and Onosu (2023)	USA	Empirical	Qualitative (narrative analysis)	Leaders
Mazzetti et al. (2022)	Italy	Empirical	Validation study (psychometric)	Employees
Njaramba (2024)	Kenya	Empirical	Cross-sectional (survey)	Employees
Nunes and Palma-Moreira (2024)	Portugal	Empirical	Cross-sectional	Employees
Palvimo et al. (2023)	Finland	Empirical	Cross-sectional	Nurses
Peng et al. (2023)	China	Empirical	Cross-sectional	Public employees
Plouffe et al. (2023)	Canada	Empirical	Longitudinal (panel)	Healthcare workers
Santiago-Torner et al. (2024b)	Spain	Empirical	Cross-sectional (SEM)	Employees
Schowalter and Volmer (2024)	Germany	Empirical	Cross-sectional	Employees
Stosic et al. (2022)	USA	Empirical	Cross-sectional (quantitative)	Professionals
Tafvelin et al. (2025)	Sweden	Empirical	Cross-sectional (quantitative)	Leaders
Vonderlin et al. (2023)	Germany	Empirical	Intervention (pre-post design)	Employees
Wang and Hannah (2025)	USA	Empirical	Cross-sectional (quantitative)	Employees
Zada et al. (2022)	Pakistan	Empirical	Cross-sectional (moderated mediation)	Employees
Zhang et al. (2022)	China	Empirical	Cross-sectional (moderated mediation)	Employees
Zheng et al. (2025)	China	Empirical	Cross-sectional	Nurses
Dirani et al. (2020)	International	Conceptual	Theoretical analysis	—
Irving (2025)	International	Conceptual	Book chapter	—
Keselman and Saxe-Braithwaite (2021)	International	Conceptual	Commentary	—
Labrague (2024)	International	Conceptual	Systematic review	Studies
Lehtonen et al. (2025)	International	Conceptual	Narrative review	Studies
P. Li et al. (2024)	International	Conceptual	Meta-analysis	Studies
Markey et al. (2021)	International	Conceptual	Theoretical discussion	—
Northouse (2025)	International	Conceptual	Book (theoretical)	—
Riggio and Newstead (2023)	International	Conceptual	Integrative review	Studies
Rudolph et al. (2020)	International	Conceptual	Systematic review	Studies
Schlütter et al. (2024)	International	Conceptual	Systematic review and critical reflection	Studies
Wu et al. (2021)	International	Conceptual	Narrative review	Studies
Wong et al. (2025)	International	Conceptual	Systematic review	Studies

**Table 2:** Frequency of leadership styles, mechanisms, and outcomes (2020–2025).

Category	Subcategory	Frequency (No. of Studies)
Leadership Styles	Transformational	6
	Servant	5
	Ethical	5
	Inclusive	1
	Health-oriented	3
	Authoritarian	3
	Toxic/Destructive	5
	Crisis/Relational/Empathic	4
Mechanisms	Psychological Safety	4
	Trust (leader/org.)	3
	Organizational Climate/Ethical Climate	8
	Psychological Capital	7
	Rumination/Worry	4
	Stress/Anxiety processes	6
Outcomes	Burnout/Emotional Exhaustion	10
	Psychological Distress	4
	Well-being/Psychological Well-being	5
	Work Engagement/Commitment	3
	Resilience	3
	Organizational Silence	1
	Turnover Intentions	3
	Job Satisfaction	3

Note. Categories are not mutually exclusive; individual sources may contribute to more than one category.

## Data Availability

All data analyzed in this scoping review derive from previously published studies cited in the references. The review protocol was registered on the Open Science Framework (OSF) and is publicly available at: https://osf.io/5xmyj/ (accessed on 17 August 2025). A PRISMA-ScR checklist is available in the Supplementary Materials.

## Supplementary Materials

The following supporting information can be downloaded at https://www.mdpi.com/article/10.3390/ejihpe16090138/s1, A PRISMA-ScR checklist. Reference Tricco et al. (2018) is cited in the Supplementary Materials.

## Abbreviations

The following abbreviations are used in this manuscript:

PRISMA-ScR	Preferred Reporting Items for Systematic Reviews and Meta-Analyses extension for Scoping Reviews
SEM	Structural Equation Modelin
OSF	Open Science Framework
JD-R	Job Demands–Resources
COR	Conservation of Resources
BAT-12	Burnout Assessment Tool (12-item version)

## References

[B1-ejihpe-16-00138] Abolnasser M. S. A., Abdou A. H., Hassan T. H., Salem A. E. (2023). Transformational leadership, employee engagement, job satisfaction, and psychological well-being among hotel employees after the height of the COVID-19 pandemic: A serial mediation model. International Journal of Environmental Research and Public Health.

[B2-ejihpe-16-00138] Ahmed F., Xiong Z., Faraz N. A., Arslan A. (2023). The interplay between servant leadership, psychological safety, trust in a leader and burnout: Assessing causal relationships through a three-wave longitudinal study. International Journal of Occupational Safety and Ergonomics.

[B3-ejihpe-16-00138] Alhaidan H. (2025). Ethical leadership in action: Understanding the mechanism of organizational justice and leaders’ moral identity. Human Systems Management.

[B4-ejihpe-16-00138] Alvesson M., Sandberg J. (2011). Generating research questions through problematization. Academy of Management Review.

[B5-ejihpe-16-00138] Anastasiou S. (2025). Counteracting toxic leadership in education: Transforming schools through emotional intelligence and ethical leadership. Administrative Sciences.

[B6-ejihpe-16-00138] Asim M., Zhiying L., Nadeem M. A., Ghani U., Arshad M., Yi X. (2021). How authoritarian leadership affects employee’s helping behavior? The mediating role of rumination and moderating role of psychological ownership. Frontiers in Psychology.

[B7-ejihpe-16-00138] Aslan M., Sönmez S., Deniz M. (2025). Leadership styles and work stress: The role of workplace climate and feelings of entrapment. Current Psychology.

[B8-ejihpe-16-00138] Bakker A. B., Demerouti E. (2017). Job demands–resources theory: Taking stock and looking forward. Journal of Occupational Health Psychology.

[B9-ejihpe-16-00138] De Clercq D., Belausteguigoitia I. (2021). The links among interpersonal conflict, personal and contextual resources, and creative behaviour. Canadian Journal of Administrative Sciences/Revue Canadienne des Sciences de l’Administration.

[B10-ejihpe-16-00138] Dirani K. M., Abadi M., Alizadeh A., Barhate B., Garza R. C., Gunasekara N., Ibrahim G., Majzun Z. (2020). Leadership competencies and the essential role of human resource development in times of crisis: A response to COVID-19 pandemic. Human Resource Development International.

[B11-ejihpe-16-00138] Ertem H. Y. (2024). School leadership fostering mental health in the times of crisis: Synthesis of school principals’ views and PISA 2022. BMC Psychology.

[B12-ejihpe-16-00138] Fajardo Castro L. V., Camargo Escobar I. M., Manrique Torres A. M., León Rincón D., Jiménez Carranza C. C., Aguilar Bustamante M. C. (2025). Liderazgo transformacional, clima organizacional y el papel mediador de la seguridad psicológica. Acta Colombiana de Psicología.

[B13-ejihpe-16-00138] Farghaly Abdelaliem S. M., Abou Zeid M. A. G. (2023). The relationship between toxic leadership and organizational performance: The mediating effect of nurses’ silence. BMC Nursing.

[B14-ejihpe-16-00138] Ferrari R. (2015). Writing narrative style literature reviews. Medical Writing.

[B15-ejihpe-16-00138] Gilson L. L., Goldberg C. B. (2015). Editors’ comment: So, what is a conceptual paper?. Group & Organization Management.

[B16-ejihpe-16-00138] Greenhalgh T., Thorne S., Malterud K. (2018). Time to challenge the spurious hierarchy of systematic over narrative reviews?. European Journal of Clinical Investigation.

[B17-ejihpe-16-00138] Haddaway N. R., Page M. J., Pritchard C. C., McGuinness L. A. (2022). PRISMA2020: An R package and Shiny app for producing PRISMA 2020-compliant flow diagrams, with interactivity for optimised digital transparency and Open Synthesis. Campbell Systematic Reviews.

[B18-ejihpe-16-00138] Hobfoll S. E., Halbesleben J., Neveu J.-P., Westman M. (2018). Conservation of resources in the organizational context: The reality of resources and their consequences. Annual Review of Organizational Psychology and Organizational Behavior.

[B19-ejihpe-16-00138] Irving J. A. (2025). Cross-cultural perspectives on servant leadership. Servant leadership: Developments in theory and research.

[B20-ejihpe-16-00138] Keselman D., Saxe-Braithwaite M. (2021). Authentic and ethical leadership during a crisis. Healthcare Management Forum.

[B21-ejihpe-16-00138] Khan H. S., Rehmat M., Butt T. H., Farooqi S., Asim J. (2020). Impact of transformational leadership on work performance, burnout and social loafing: A mediation model. Future Business Journal.

[B22-ejihpe-16-00138] Klebe L., Felfe J., Klug K. (2021). Healthy leadership in turbulent times: The effectiveness of health-oriented leadership in crisis. British Journal of Management.

[B23-ejihpe-16-00138] Kloutsiniotis P. V., Mihail D. M., Mylonas N., Pateli A. (2022). Transformational Leadership, HRM practices and burnout during the COVID-19 pandemic: The role of personal stress, anxiety, and workplace loneliness. International Journal of Hospitality Management.

[B24-ejihpe-16-00138] Krick A., Felfe J., Pischel S. (2022). Health-oriented leadership as a job resource: Can staff care buffer the effects of job demands on employee health and job satisfaction?. Journal of Managerial Psychology.

[B25-ejihpe-16-00138] Labrague L. J. (2024). Toxic leadership and its relationship with outcomes on the nursing workforce and patient safety: A systematic review. Leadership in Health Services.

[B26-ejihpe-16-00138] Lehtonen S., Seeck H., Satama S., Raelin J. A., Huhtinen A. M. (2025). Rethinking crisis leadership through leadership-as-practice: A narrative review and future directions. International Journal of Disaster Risk Reduction.

[B27-ejihpe-16-00138] Lester P. B., Harms P. D., DeSimone J. A. (2024). Taken to the extreme: Transformational leadership, psychological capital, and follower health outcomes in extreme contexts. Military Psychology.

[B28-ejihpe-16-00138] Li P., Yin K., Shi J., Damen T. G., Taris T. W. (2024). Are bad leaders indeed bad for employees? A meta-analysis of longitudinal studies between destructive leadership and employee outcomes. Journal of Business Ethics.

[B29-ejihpe-16-00138] Li X., Peng P. (2022). How does inclusive leadership curb workers’ emotional exhaustion? The mediation of caring ethical climate and psychological safety. Frontiers in Psychology.

[B30-ejihpe-16-00138] Linvill J. S., Onosu G. O. (2023). Stories of leadership: Leading with empathy through the COVID-19 pandemic. Sustainability.

[B31-ejihpe-16-00138] Markey K., Ventura C. A. A., Donnell C. O., Doody O. (2021). Cultivating ethical leadership in the recovery of COVID-19. Journal of Nursing Management.

[B32-ejihpe-16-00138] Mazzetti G., Consiglio C., Santarpia F. P., Borgogni L., Guglielmi D., Schaufeli W. B. (2022). Italian validation of the 12-item version of the Burnout Assessment Tool (BAT-12). International Journal of Environmental Research and Public Health.

[B33-ejihpe-16-00138] Njaramba F. (2024). Transformational leadership in a crisis: Dimensional analysis with psychological capital. Heliyon.

[B34-ejihpe-16-00138] Northouse P. G. (2025). Leadership: Theory and practice.

[B35-ejihpe-16-00138] Nunes A., Palma-Moreira A. (2024). Toxic leadership and turnover intentions: The role of burnout syndrome. Administrative Sciences.

[B36-ejihpe-16-00138] Orozco-Solis M. G., Bravo-Andrade H. R., Ruvalcaba-Romero N. A., Ángel-González M., Vázquez-Juárez C. L., Vázquez-Colunga J. C. (2022). Socialización organizacional y salud mental positiva ocupacional como predictores del compromiso organizacional en docentes de educación superior. Acta Colombiana de Psicología.

[B37-ejihpe-16-00138] Page M. J., McKenzie J. E., Bossuyt P. M., Boutron I., Hoffmann T. C., Mulrow C. D., Shamseer L., Tetzlaff J. M., Akl E. A., Brennan S. E., Chou R., Glanville J., Grimshaw J. M., Hróbjartsson A., Lalu M. M., Li T., Loder E. W., Mayo-Wilson E., McDonald S., Moher D. (2021). The PRISMA 2020 statement: An updated guideline for reporting systematic reviews. BMJ.

[B38-ejihpe-16-00138] Palvimo T., Vauhkonen A., Hult M. (2023). The associations among destructive leadership, job demands and resources, and burnout among nurses: A cross-sectional survey study. Journal of Nursing Management.

[B39-ejihpe-16-00138] Paré G., Trudel M. C., Jaana M., Kitsiou S. (2015). Synthesizing information systems knowledge: A typology of literature reviews. Information & Management.

[B40-ejihpe-16-00138] Peng C., Liang Y., Yuan G., Xie M., Mao Y., Harmat L., Bonaiuto F. (2023). How servant leadership predicts employee resilience in public organizations: A social identity perspective. Current Psychology.

[B41-ejihpe-16-00138] Plouffe R. A., Nazarov A., Heesters A. M., Dickey C. C., Foxcroft L., Hosseiny F., Le T., Lum P. A., Nouri M. S., Smith P., Richardson J. D. (2023). The mediating roles of workplace support and ethical work environment in associations between leadership and moral distress: A longitudinal study of Canadian health care workers during the COVID-19 pandemic. Frontiers in Psychology.

[B42-ejihpe-16-00138] Riggio R. E., Newstead T. (2023). Crisis leadership. Annual Review of Organizational Psychology and Organizational Behavior.

[B43-ejihpe-16-00138] Rudolph C. W., Murphy L. D., Zacher H. (2020). A systematic review and critique of research on “healthy leadership”. The Leadership Quarterly.

[B44-ejihpe-16-00138] Santiago-Torner C. (2023). Selfish ethical climate and teleworking in the Colombian electricity sector. The moderating role of ethical leadership. Acta Colombiana de Psicología.

[B45-ejihpe-16-00138] Santiago-Torner C., Corral-Marfil J. A., Tarrats-Pons E. (2024a). The Relationship Between Ethical Leadership and Emotional Exhaustion in a Virtual Work Environment: A Moderated Mediation Model. Systems.

[B46-ejihpe-16-00138] Santiago-Torner C., González-Carrasco M., Miranda Ayala R. A. (2024b). Ethical leadership and emotional exhaustion: The impact of moral intensity and affective commitment. Administrative Sciences.

[B47-ejihpe-16-00138] Santiago-Torner C., Jiménez-Pérez Y., Tarrats-Pons E. (2025). Interaction between ethical leadership, affective commitment and social sustainability in transition economies: A model mediated by ethical climate and moderated by psychological empowerment in the Colombian electricity sector. Sustainability.

[B48-ejihpe-16-00138] Schlütter D., Schätzlein L., Hahn R., Waldner C. (2024). Missing the impact in impact investing research–a systematic review and critical reflection of the literature. Journal of Management Studies.

[B49-ejihpe-16-00138] Schowalter A. F., Volmer J. (2024). Servant and Crisis Manager? The Association of Servant Leadership with Followers’ Adaptivity and Proactivity. Journal of Leadership & Organizational Studies.

[B50-ejihpe-16-00138] Stosic M. D., Blanch-Hartigan D., Aleksanyan T., Duenas J., Ruben M. A. (2022). Empathy, friend or foe? Untangling the relationship between empathy and burnout in helping professions. The Journal of Social Psychology.

[B51-ejihpe-16-00138] Tafvelin S., Irehill H., Lundmark R. (2025). Are young leaders more sensitive to contextual influences? A lifespan perspective on organizational antecedents of transformational leadership. Nordic Psychology.

[B52-ejihpe-16-00138] Templier M., Paré G. (2015). A framework for guiding and evaluating literature reviews. Communications of the Association for Information Systems.

[B53-ejihpe-16-00138] Tricco A. C., Lillie E., Zarin W., O’Brien K. K., Colquhoun H., Levac D., Moher D., Peters M. D. J., Horsley T., Weeks L., Hempel S., Akl E. A., Chang C., McGowan J., Stewart L., Hartling L., Aldcroft A., Wilson M. G., Garritty C., Straus S. E. (2018). PRISMA Extension for scoping reviews (PRISMA-ScR): Checklist and explanation. Annals of Internal Medicine.

[B54-ejihpe-16-00138] Vonderlin R., Schmidt B., Biermann M., Lyssenko L., Heinzel-Gutenbrunner M., Kleindienst N., Bohus M., Müller G. (2023). Improving health and reducing absence days at work: Effects of a mindfulness-and skill-based leadership intervention on supervisor and employee sick days. Mindfulness.

[B55-ejihpe-16-00138] Wang Z., Hannah S. T. (2025). Organizational ethical pressure as a threat to employee health: The buffering roles of ethical leadership and employee ethical efficacy. Business Ethics, the Environment & Responsibility.

[B56-ejihpe-16-00138] Wong A. K. F., Kim S., Gamor E., Koseoglu M. A., Liu Y. (2025). Advancing employees’ mental health and psychological well-being research in hospitality and tourism: Systematic review, critical reflections, and future prospects. Journal of Hospitality & Tourism Research.

[B57-ejihpe-16-00138] Wu Y. L., Shao B., Newman A., Schwarz G. (2021). Crisis leadership: A review and future research agenda. The Leadership Quarterly.

[B58-ejihpe-16-00138] Zada M., Zada S., Khan J., Saeed I., Zhang Y. J., Vega-Muñoz A., Salazar-Sepúlveda G. (2022). Does servant leadership control psychological distress in crisis? Moderation and mediation mechanism. Psychology Research and Behavior Management.

[B59-ejihpe-16-00138] Zhang Y., Wang J., Akhtar M. N., Wang Y. (2022). Authoritarian leadership and cyberloafing: A moderated mediation model of emotional exhaustion and power distance orientation. Frontiers in Psychology.

[B60-ejihpe-16-00138] Zheng X., Song J., Shi X., Kan C., Chen C. (2025). The effect of authoritarian leadership on young nurses’ burnout: The mediating role of organizational climate and psychological capital. BMC Health Services Research.

